# Measuring Software Modularity Based on Software Networks

**DOI:** 10.3390/e21040344

**Published:** 2019-03-28

**Authors:** Yiming Xiang, Weifeng Pan, Haibo Jiang, Yunfang Zhu, Hao Li

**Affiliations:** 1 School of Management and E-Business, Zhejiang Gongshang University, Hangzhou 310018, China; 2 School of Computer Science and Information Engineering, Zhejiang Gongshang University, Hangzhou 310018, China; 3 Department of Computer Science, Western Michigan University, Kalamazoo, MI 49008, USA

**Keywords:** modularity, Java software, complex networks, software metrics, software maintenance

## Abstract

Modularity has been regarded as one of the most important properties of a successful software design. It has significant impact on many external quality attributes such as reusability, maintainability, and understandability. Thus, proposing metrics to measure the software modularity can be very useful. Although several metrics have been proposed to characterize some modularity-related attributes, they fail to characterize software modularity as a whole. A complex network uses network models to abstract the internal structure of complex systems, providing a general way to analyze complex systems as a whole. In this paper, we introduce the complex network theory into software engineering and employ *modularity*, a metric widely used in the field of community detection in complex network research, to measure software modularity as a whole. First, a specific piece of software is represented by a software network, feature coupling network (FCN), where methods and attributes are nodes, couplings between methods and attributes are edges, and the weight on the edges denotes the coupling strength. Then, *modularity* is applied to the FCN to measure software modularity. We apply the Weyuker’s criteria which is widely used in the field of software metrics, to validate the *modularity* as a software metric theoretically, and also perform an empirical evaluation using open-source Java software systems to show its effectiveness as a software metric to measure software modularity.

## 1. Introduction

“High cohesion and low coupling” is one of the most important principles in object-oriented (OO) designs [1]. ‘Cohesion’ is the indication of the coupling within a module, while ‘coupling’ is the indication of the coupling between modules. When designing a piece of software, we usually strive for high cohesion (a cohesive module) and low coupling (couplings between modules should be less), which promotes the formation of a modular structure in a piece of software. Modularity has been regarded as one of the most important properties of a software design, which has significant impact on many external quality attributes such as reusability, maintainability, and understandability. By saying modularity, it usually means the notion of interdependence within modules and independence between modules [2].

We cannot control what we cannot measure [3]. Thus, to control software modularity, we need quantitative techniques to assess it. One of the effective techniques is to provide some metrics to characterize modularity-related attributes such as module coupling, cohesion and interface size [4]. However, many of these metrics only focus on one aspect of modularity, either coupling or cohesion. They cannot take into consideration both the coupling and cohesion to character software modularity, and cannot draw a whole picture of the software modularity. Worse still, they are usually used to characterize some local features of a piece of software, e.g., the number of classes that a class couples with, the number of methods that a class has, etc. They failed to explore the rich information in the whole structure of a specific software system. Due to a lack of suitable tools and theories, people seldom characterize the modularity of a specific software system as a whole.

As we all know, any software system is composed of a set of software elements (e.g., methods, attributes, classes, etc.) and their couplings (e.g., “method call”, “inheritance”, “extends”, etc.), which constitute the topological structure of the software [5]. The software structure of a specific piece of software is formed in the whole production process of the software and can be naturally represented by a network (or a graph). In recent years, many attempts have been made to employ complex network theories to analyze software structures and their dynamics by a network representation (usually named software networks) of the software structure, where software elements are nodes, and their couplings are edges [5,6,7,8]. They found that a software network is a complex network that has non-trivial topological features that are not observed in simple networks such as lattices or random graphs. Their work opens up an interdisciplinary research between complex networks and software engineering, and many physics-like laws of software networks have been revealed such as “scale-free” and “small-world” [5,6,7,8]. From the point of view of entropy, software structure changes from chaos to order in the whole production process [9,10]. Furthermore, complex network theories have also been applied to refactor software [11], identify important software elements such as important packages and classes [12], and prioritize test cases [13]. Such a novel interdisciplinary research provides us with an effective tool to analyze software as a whole.

In this paper, our aim is to characterize software modularity as a whole using complex network theories. To fulfill this task, we first propose a feature coupling network (FCN) to represent the software structure at the method and attribute level, where methods and attributes are nodes, couplings between methods and attributes are edges, and the weight on the edges denotes the coupling strength. Then, we employ *modularity*, a metric widely used in the field of community detection in complex network research, to measure software modularity as a whole. We apply the widely accepted Weyuker’s criteria [14] to validate our *modularity* theoretically, and also perform an empirical evaluation using open-source Java software systems to show its effectiveness as a metric to measure software modularity. Note that our approach now can only be applied to software developed using Java since our own developed software now can only parse Java software.

The main contributions of this work can be summarized as follows:

- We characterize software modularity as a whole. The existing metrics usually only characterized some modularity-related attributes—either coupling or cohesion. They cannot take both the coupling and cohesion into consideration to character software modularity. In this work, we use software networks to represent software, and apply the metric *modularity* in complex network research to character software modularity. Thus, we can characterize software modularity as a whole.
- The proposed metric *modularity* considers the coupling strength between software elements which has been neglected by the existing metrics. Our proposed software network, FCN, is a weighted software network. The weight on the edges denotes the coupling strength between software elements. The calculation of *modularity* considers the weight on the edge. Thus, our metric is more reasonable since it conforms to the reality of a specific piece of software.
- The proposed *modularity* is validated theoretically using widely accepted evaluation criteria, and empirically using open source Java software systems. The data set and software used to compute software modularity are available for download [15] (see in Supplementary Materials).

The rest of this paper is organized as follows. Section 2 reviews the related work. Section 3 describes our approach in detail, with a focus on the definition of FCN and the software modularity metric. Section 4 presents the theoretical and empirical validations of our proposed metric. We conclude in Section 5.

## 2. Related Work

The existing research work to measure software modularity mainly focuses on measuring the coupling between software elements and the cohesion of software elements. In this section, we summarize the existing metrics on measuring coupling and cohesion at different levels of granularity. Generally, cohesion measurement should rely on the coupling measurement.

Chidamber and Kemerer [16] proposed Lack of Cohesion in Methods (LCOM) to measure class cohesion. Eder et al. [17] defined three types of cohesion in OO software systems, i.e., method cohesion, class cohesion, and inheritance cohesion. Ott et al. [18] defined a class cohesion metric based on data slice for OO software systems. Bieman and Kang [19] defined two class cohesion metrics, Tight Class Cohesion (TCC) and Loose Class Cohesion (LCC), to measure class cohesion by counting the number of pairs of methods in a class that access common attributes. Bansiya and Davis [20] defined a metric, Cohesion Among Methods of Classes (CAMC), to measure class cohesion by quantifying the similarity of methods according to their parameter lists. Briand et al. [21] applied an interaction graph to represent software structure, and proposed a suite of cohesion metrics. Morris [22] measured module cohesion by quantifying the fan-in of objects that the module contains. Patel et al. [23] proposed a metric to measure subprogram cohesion by quantifying the similarity between different pairs of subprograms. Martin [24] defined a metric, Relational Cohesion (RC), to measure package cohesion by using the ratio of the number of data relations and number of components in a package. Lee and Liang [25] defined a metric, Information-based CoHesion (ICH) based on information flow. Chidamber and Kemerer [16] proposed a CK (Chidamber & Kemerer) metric suite to measure software couplings. Jenkins et al. [26] defined a metric, $I_{cc}$, to measure software stability based on the classes and their couplings. Qu et al. [27] explored the community structure in software systems and measured class cohesion using community detection techniques. Gu et al. [28] proposed a new metric to measure class cohesion based on computing the average clustering coefficient of software network at the class level. Zhang et al. [29] applied community detection techniques to measure the cohesion of a software system.

However, most of these metrics focus mainly on one aspect of modularity, either coupling or cohesion. They neglect to consider both of the coupling and cohesion to measure software modularity, cannot drawing a whole picture of the software modularity. Worse still, they mainly focus on the characterization of some local features of a piece of software. There is a need for metrics to characterize the software modularity as a whole.

## 3. The Proposed Approach

**Figure 1:**
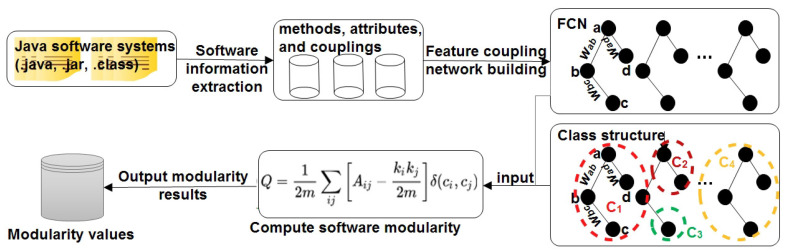
An overview of the proposed approach.

Figure 1 shows an overview of our approach to measure software modularity using complex network theories. Our approach is mainly composed of three steps. First, we extract software structural information from the source code of a software system by static analysis. Second, we propose the FCN to represent the extracted software structural information. Finally, we apply the *modularity* metric widely used in complex network theories to measure software modularity as a whole. We will detail the three parts in the following subsections.

### 3.1. Software Information Extraction

In this work, we choose to analyze software systems coded in Java simply for our own developed analysis software, SNAP [12], now can only analyze software systems coded in Java, and Java is one of the most successful and popular programming languages.

As mentioned above, we use a software network to represent software elements and their couplings at the method and attribute level. Thus, software elements and their couplings in a specific Java software system should be extracted first. To this aim, we perform a static analysis of the source code of the software and extract structural information at the feature (In this work, if not mentioned, the term “feature” designates both the methods and attributes from here on) level, i.e., we extract methods, attributes, and their couplings. Here, we consider two types of couplings between features, i.e., “method-call” couplings and “method-access-attribute” couplings.

Note that, when computing the software modularity, we should refer to the class structure of the software. Thus, we also extract class information, i.e., we extract all classes, and the methods and attributes they contain. It should also be noted that we only consider the software elements that are actually defined in the software. For those classes that are defined in the imported libraries will be ignored, since their source codes are not always available in the source code distribution of the software [12].

### 3.2. Feature Coupling Network

The structural information obtained in Section 3.1 will be further represented by FCN, which is defined as follows.

**Definition** **1** (FCN)**.** *FCN is a weighted undirected graph representing features (methods and attributes) and their couplings in a specific software system. Specifically, nodes in FCN denote the methods and attributes in the software, and edges in FCN denote the coupling between methods and attributes, i.e., “method-call” couplings and “method-access-attribute” couplings. The weight on the edge denotes the multiplicity of couplings such as method* $m_{1}$ *calls method* $m_{2}$ *three times. Note that each method or attribute is represented by only one node. Thus, FCN can be defined as*

(1) FCN=(N,E,ψ),

*where N is the node set, E is the edge set, and* ψ *is a symmetric matrix storing the weight on the edge between all pairs of nodes if they are linked together by an edge in FCN. Specifically, if method **i** couples with method **j**, the entries* $\psi_{\mathrm{ij}}$ *and* $\psi_{\mathrm{ji}}$ *(* $\psi_{\mathrm{ij}}$ *=* $\psi_{\mathrm{ji}}$ *) of* ψ *stores the weight on the edge between method **i** and method **j**. The weight on the edge provides us a more accurate representation of the software structure at the feature level and can be obtained by simply counting their occurrences in our extracted structural information (see Section 3.1).*

**Figure 2:**
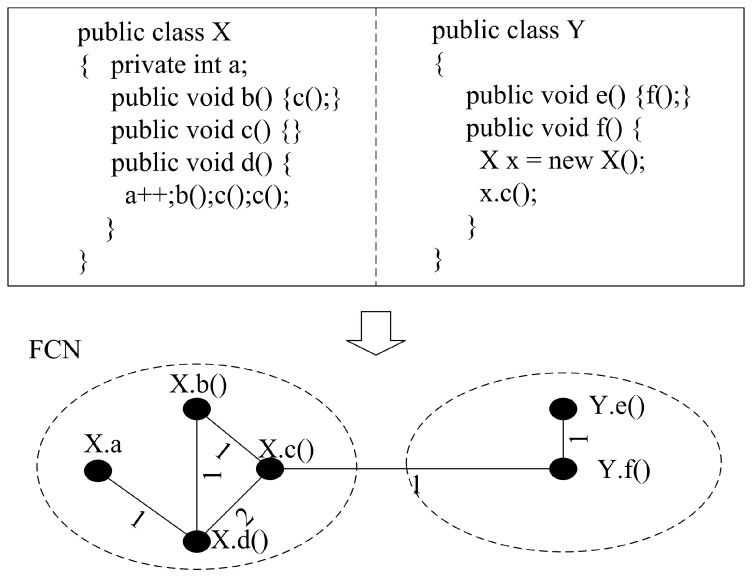
A simple code segment (**top**) and its corresponding FCN (Feature Coupling Network) (**bottom**).

Figure 2 gives a simple example to show the process to build FCN from Java source code. The notes beside the nodes denote the method name or attribute name that the node denotes, and the notes beside the edges denote the weight on that edge. Since method “d()” accesses attribute “a” one time, calls method “b()” one time, and calls method “c()” two times, there are three edges between “d()” and “a”, “d()” and “b()”, and “d()” and “c()”, with the weights being 1, 1, and 2, respectively. Since method “b()” calls method “c()” one time, there is one edge between “b()” and “c()” with weight being 1. Since method “f()” calls method “c()” one time, there is one edge between “f()” and “c()” with the weight being 1. Since method “e()” calls method “f()” one time, there is one edge between “e()” and “f()” with the weight being 1.

### 3.3. Software Modularity

In software engineering, developers are advocated to incorporate related attributes and methods into modules, and reduce the coupling between modules. The “module" in software is very similar to the concept “community" in complex network research. In complex networks, communities are subsets of densely connected nodes such there is a higher density of edges in the community than between communities. Community structure has become one of the most important network properties that can be observed in many networked systems. Software systems also have community structures when representing software systems as software networks [11,30]. Generally, packages are the natural communities of classes and interfaces, and classes and interfaces are the natural communities of methods and attributes [30]. Thus, we can use the quality index that is used to quantify the community structure in complex networks to measure software modularity.

In complex networks, many quality indexes have been proposed to evaluate the community structure such as MQ [31], EVM [32], and *modularity* (*Q*) [33]. Arguably, *Q* proposed by Newman and Girvan is the most widely used and famous quality index. It is also used to measure the density of edges within communities compared with edges between communities. In this work, we also use *Q* to compute the software modularity. For a weighted undirected network, our *modularity* metric can be defined as

(2) $$Q=\frac{1}{2m}\sum_{ij}\left[A_{ij}-\frac{k_{i}k_{j}}{2m}\right]\delta \left(c_{i},c_{j}\right),$$

where *m* is the sum of the weights on all the edges in the network, $A_{ij}$ is the weight on the edge between nodes *i* and *j*, $k_{i}$ and $k_{j}$ are the sum of the weights on the edges attached to nodes *i* and *j*, respectively, $c_{i}$ and $c_{j}$ are the communities that nodes *i* and *j* belong to, respectively, and δ is a simple delta function that takes 1 when $c_{i}$ equals $c_{j}$, 0, otherwise.

Obviously, we can observe from Equation (2) that δ function makes sure a coupling between two nodes from two different communities makes no contribution to *Q*. Two nodes linked by one edge in a community make a positive contribution to *Q* while two isolated nodes in a community provide a negative contribution to *Q*.

Generally, a higher modularity value denotes a more reasonable community structure where nodes in the community densely coupled with each other than between communities. When computing *Q* of FCN, we take the class or interface structure in the software as the nature community of methods and attributes, i.e., methods and attributes defined in the same class or interface belong to the same community. Then, by using *Q* to FCN, we can obtain the software modularity, which is a measurement of the degree of the “high cohesion and low coupling" that the software adheres to.

### 3.4. Pseudo-Code of the Algorithm to Compute *Q*

Algorithm 1 shows the pseudo-code of the algorithm that we used to compute *Q*, where *k* is an array used to store the the sum of the weights on the edges attached to node *i*, and getClass(i) is a function used to return the class that feature *i* is defined in.
**Algorithm 1** Pseudo-code of the algorithm to compute *Q***Input:**  FCN, |N| (number of nodes in FCN), and *A* (ψ in FCN).**Output:**  Print the *Q*.  1:sum_2m = 0, sum_sigma = 0;  2:**for**i=1 to |N| **do**  3:  k[i] = 0; //$k_{i}$  4:  **for** j=1 to |N| **do**  5:    sum_2m += A[i][j]; //2m  6:    **if** A[i][j]>0 **then**
  7:      k[i]+=A[i][j]  8:    **end if**  9:  **end for**10:**end for**11:**for**i=1 to |N| **do**12:  **for** j=1 to |N| **do**13:    **if** getClass(i)==getClass(j) **then**14:      sum_sigma+=A[i][j]-k[i]*k[j]/sum_2m;15:    **end if**16:  **end for**17:**end for**18:Q=sum_sigma/sum_2m;19:print *Q*;

## 4. Evaluations

In this section, we validated our software modularity metric theoretically using the widely accept criteria, and also empirically evaluated the metric using a set of Java software systems. Our empirical experiments were carried out on a ThinkPad E420S machine with Window 7 OS, a 2.30 GHz Intel Core i5-2410M CPU, and 6 GB RAM.

In the following sections, we list the research questions that we focus on (Section 4.1), and our answers to the research questions (Section 4.2).

### 4.1. Research Questions

In this work, our evaluations aimed at addressing the following four research questions (RQs):

- RQ1: Does *Q* satisfy Weyuker’s nine properties? *Q* is a metric used to measure software modularity and also can be classified into the category of complexity metrics. Weyuker’s nine properties are widely used and famous criteria to validate the usefulness of software complex metrics. We wish to know whether our *Q* also satisfies Weyuker’s nine properties.
- RQ2: What about the *Q* values obtained in different software systems? Different software systems may have different *Q* values. For interests, we wish to examine the *Q* values obtained in different software systems.
- RQ3: Can *Q* tell the software using design patterns from two function-equivalent software systems? Using design patterns in software development is regarded as an effective way to improve software quality. However, design pattern implementations may suffer from some of the typical problems and heavily affect the software modularity. As an effective metric, *Q* should have the ability to reflect such a degradation in software modularity. Thus, we wish to know whether our *Q* has the ability to tell the software using design pattern from two function-equivalent software systems (one uses design patterns, and the other not).
- RQ4: Is *Q* scalable to large software systems? In practise, a software metric will be applied to software systems with different sizes. Thus, we wish to know whether *Q* can be applied to larger software systems.

### 4.2. Answers to Research Questions

In this section, we performed theoretical analysis and empirical experiments to answer the RQs raised in Section 4.1.

#### 4.2.1. RQ1: Does *Q* Satisfy Weyuker’s Nine Properties?

Weyuker’s nine properties are the widely used and most famous criteria to evaluate the efficiency and robustness of any software complexity metric [14]. It is a theoretical framework that is designed to check whether a metric is qualified as an effective metric. In this section, we also validate our *Q* using Weyuker’s nine properties property-by-property. In the following paragraphs, *M* denotes any software complexity metric. In this work, *M* refers to *Q*.

**Property** **1** (Non-coarseness)**.** (∃P)(∃E)(M(P)≠M(E)) *, where P and E are two different programs.*

**Proof.**  Two different Java software systems *P* and *E* usually have different feature sets and coupling sets (couplings between features). Furthermore, their class structures may also be different. Thus, we can assume that the FCNs built from the two software systems may be different, which results in different *Q* values for the two software systems. Therefore, *Q* does adhere to Property 1. □

**Property** **2** (Granularity)**.** *Let c be a non-negative number; then, there are only finitely many programs P with*M(P)=c.

**Proof.**  Since the universe of discourse deals with a finite number of applications. Thus, there are only a finite amount of software systems with the same FCNs and class structures which satisfy Q=c. Therefore, *Q* does adhere to Property 2. □

**Property** **3** (Non-uniqueness)**.** *There are two different programs P and E such that*M(P)=M(E).

**Proof.**  A large number of software systems have been developed and deployed. It is a reasonable assumption that there might exist two software systems with a same FCN and class structure. Therefore, *Q* does adhere to Property 3. □

**Property** **4** (Design Details are Important)**.** (∃P)(∃E)(P≡E&M(P)≠M(E)).

**Proof.**  There are many function-equivalent software systems with different FCNs and class structures. Thus, their *Q* values are different. For example, in Table 3, we list two different versions of software with the same set of functionalities. Obviously, the two different versions have different *Q* values. Therefore, *Q* does adhere to Property 4. □

**Property** **5** (Monotonicity)**.** (∃P)(∃E)(M(P)≤M(P+E)&M(E)≤M(P+E)).

**Proof.**  This property is originally proposed to check size-related metrics. Our *Q* metric is not a size-related metric. Therefore, Property 5 is not applicable to evaluate our *Q* metric. □

**Property** **6** (Non-equivalence of Interaction)**.** (∃P)(∃E)(∃R)(M(P)=M(E))&(M(P+R)=M(E+R)).

**Proof.**  *P* and *E* are two different software systems satisfying M(P)=M(E). *R* is another software program that can be correctly combined with *P* and *E*. Though the combination of *P* and *R* may produce a different FCN when compared with the combination of *E* and *R*, their *Q* values may be same. For example, suppose that *R* is a very simple software only with one method defined in one class. The combination of *P* and *R* only adds one isolated node to the FCN of *P*, which will not affect the *Q* value of *P*. Similarly, the combination of *E* and *R* also only adds one isolated node to the FCN of *E*, which will not affect the *Q* value of *E*. Thus, M(P)=M(E). Therefore, Property 6 is satisfied by *Q*. □

**Property** **7** (Significance of Permutation)**.** *For two programs, P and E (E are formed by permuting the order of the statements of P), and it can be found such that*M(P)≠M(E).

**Proof.**  This property is originally proposed for procedure-oriented metrics, and does not hold for OO metrics. Therefore, Property 7 is not satisfied by *Q*. □

**Property** **8** (No Change on Renaming)**.** *If P is a renaming of E, then*M(P)=M(E).

**Proof.**  As FCN and class structures are independent of the name of software, *Q* satisfies Property 8. □

**Property** **9** (Interaction Increases Complexity)**.** (∃P)(∃E)(M(P)+M(E)<M(P+E)).

**Proof.**  Suppose *P* and *E* are two very simple software systems only with one method defined in one class; then, we can obtain their *Q* values being 0, i.e., M(P)=M(E)=0. It is a reasonable assumption that combining *P* and *E* may result in a new FCN that has edges linking the two nodes together, making Q>0, i.e., M(P+E)>0. Therefore, *Q* satisfies Property 9. □

To sum up, our *Q* metric passes the examination of a large part (7/9) of the Weyuker’s properties, only with two exceptions, i.e., Properties 5 and 7. As mentioned above, Property 5 is not applicable to *Q* since it is proposed for size-related metrics, and our *Q* is not a size-related metric. Property 7 is not applicable to *Q* since it is proposed for procedure-oriented metrics, and our metric is an OO metric. These exceptions have also been observed in other work [16,34,35,36]. Therefore, our *Q* metric is a well-structured metric. It can be used to compute software modularity as a whole.

#### 4.2.2. RQ2: What about the *Q* Values Obtained in Different Software Systems?

Different software systems usually have different *Q* values. In this section, we performed experiments to examine the *Q* values obtained in different software systems.

(1) Subject Systems

We randomly chose a set of twelve Java software systems (see Table 1) to show the *Q* values obtained in different software systems. These systems are open-source and can be downloaded from their websites. Table 1 provides the basic information of the subject software systems, including their names, the domains that they belong to, the directory of the source code distribution that we analyzed, KLOC (thousand lines of code), and the URLs to download the corresponding software system. Without loss of generality, our subject software systems differ in size from each other, with the smallest KLOC being 2.705 and the largest KLOC being 97.880. Note that the KLOC counts the practical lines of code in the software. It does not include the comment lines and blank lines.

(2) Experiment Process and Results Analysis 

**Figure 3:**
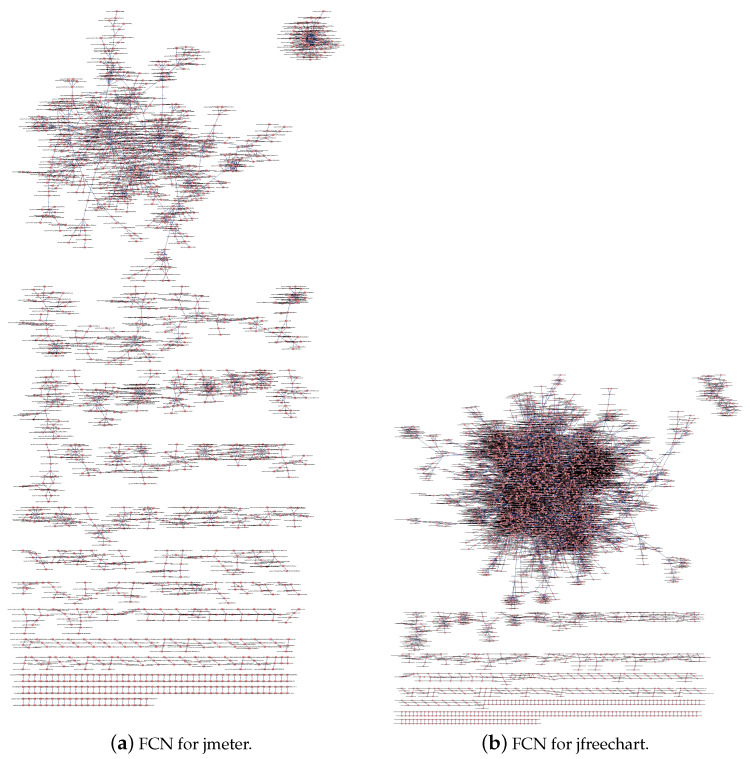
FCNs for jemter and jfreechart.

According to the steps shown in Figure 1, we analyze the source code, extract the structural information, and build the FCNs for the twelve software systems. For illustration purposes, we show the FCNs for the subject software systems jmeter and jfreechart in Figure 3. Enlarging the corresponding figure can give you the details of the figure such as the feature name that each node denotes, the edge that exists between some pairs of features, and the weight on each edge.

Table 2 shows the |N| (number of nodes), |E| (number of edges), and *Q* of the FCN of the corresponding software system. Obviously, a large part (8/12) of the subject software systems have a relative small value of *Q* with values 0.2<Q<0.4. Only four software systems have a *Q* value larger than 0.4 and smaller than 0.6.

#### 4.2.3. RQ3: Can *Q* Tell the Software Using Design Patterns from Two Function-Equivalent Software Systems?

Using design patterns in software development is regarded as an effective way to improve software quality [37]. However, design pattern implementations may suffer from some of the typical problems and heavily affect the software modularity [38]. Thus, it is reasonable to assume that *Q* can be used to tell the software using design pattern from two function-equivalent software systems (one uses design patterns, and the other not).

(1) Subject Systems

We chose five simple software systems (see Table 3), each of which has two function-equivalent versions. One version (“before” for short) does not apply any design pattern, and the other (“after” for short) applies one design pattern. The design pattern each software used is the same as the name of the software. Table 3 provides the basic information of the subject software systems, including their names, LOC (lines of code), and |N| and |E| of the FCN of the corresponding software system. Note that the LOC counts the practical lines of code in the software. It does not include the comment lines and blank lines.

(2) Experiment Process and Results Analysis

**Figure 4:**
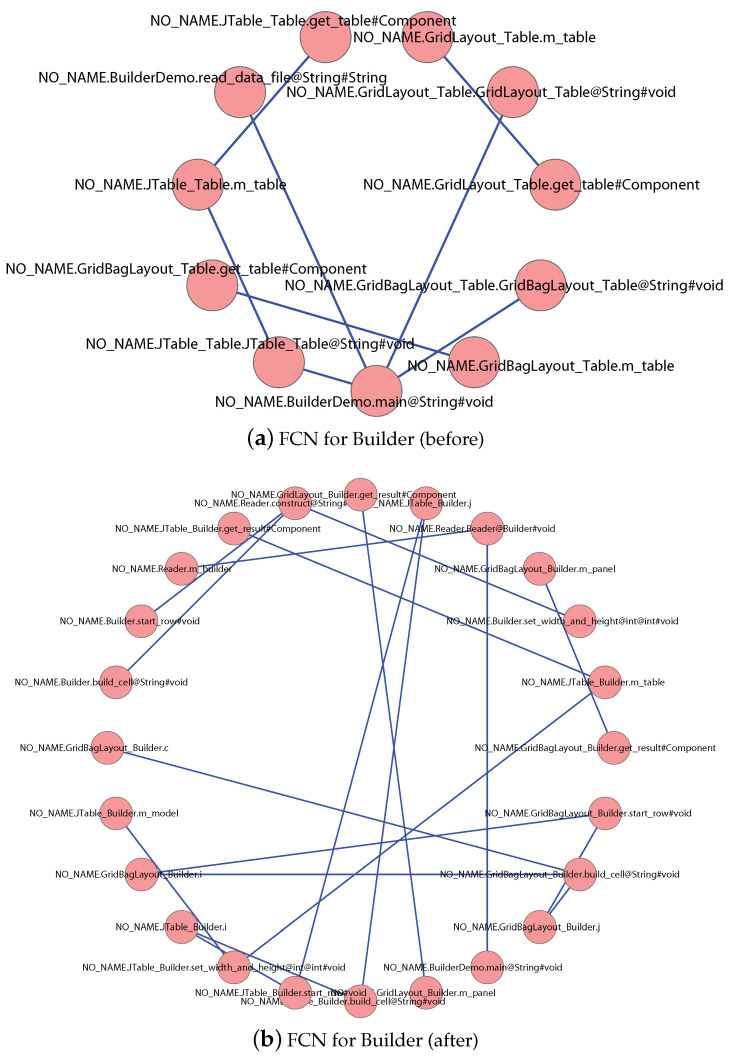
FCNs for the software Builder before and after applying the “Builder” design pattern.

According to the steps shown in Figure 1, we analyze the source code, extract the structural information, and build the FCNs for the five software systems. For illustration purposes, we show the FCNs for software “Builder” before and after applying the “Builder” design pattern in Figure 4. Enlarge the figures can give you the details of the figures such as the feature name that each node denotes, the edge that exists between some pairs of features, and the weight on each edge.

Table 3 also shows the *Q* values that we computed from the five systems. Obviously, the *Q* value for the software using design patterns is smaller than that of software that does not use design patterns. It confirms to our assumption that design pattern implementations indeed will affect software modularity.

#### 4.2.4. RQ4: Is *Q* Scalable to Large Software Systems?

In practise, a software metric will be applied to software systems with different sizes. Thus, we wish to know whether *Q* can be applied to larger software systems. To this aim, we track the execution time of each main step to evaluate the scalability of *Q*. As mentioned in Section 3, our approach is mainly composed of three steps:

(i) Extracting structural information from the source code of software systems.
(ii) Building FCNs for software systems.
(iii) Computing the software modularity according to Equation (2).

In Table 4, we show the CPU time that is required to execute each step of our approach when applied to subject software systems we chose in Section 4.2.2. We can observe that step (i) is the most time-consuming step of our approach, and the other two steps take less than one second. Though jfreechart and ant are large in size with the number of features being 11,946 and 11,858, respectively, the total CUP time used to compute *Q* is less than one minute. Thus, our approach can be scalable to large software systems. It is the answer to RQ4.

## 5. Conclusions

In this paper, we defined a novel metric, *modularity* (*Q*), to measure software modularity from the perspective of software as a whole. Our metric is based on a network representation (i.e., FCN) of the software structure at the method and attribute level, and applied the metric (i.e., *Q*) widely used in complex network theories to compute the value of software modularity. FCN is a weighted undirected software network, which considers the coupling frequencies between methods and attributes to assign weights on the edges.

Our metric is evaluated theoretically using widely accepted criteria, and empirically using open source software systems. The results show the effectiveness of *Q* as a metric to measure software modularity.

## Figures and Tables

**Table 1:** Subject software systems.

Systems	Domain	Directory	KLOC	URLs
jmeter-3.0	testing	src/core	37.951	https://jmeter.apache.org/
jfreechart-1.0.19	tool	source	97.880	http://www.jfree.org/
ant-1.6.1	parsers	src/main	81.515	https://ant.apache.org/
struts-2.5.2	middleware	src/core	48.347	https://struts.apache.org/
freemind-1.0.1	data visualization	src	45.049	http://freemind.sourceforge.net/
bcel-6.0	programming language	src	29.206	https://commons.apache.org/
mybatis-3	middleware	src	20.385	http://www.mybatis.org
colt-1.2.0	sdk	src	34.709	https://dst.lbl.gov/ACSSoftware/
jbullet-2.72.2.4	middleware	com	22.297	http://jbullet.advel.cz/
junit4-r4.12	testing	src	9.296	https://junit.org/junit4/
jxtaim-0.1i	communications	src	12.423	http://jxtaim.sourceforge.net/
commons-email-1.4	communications	src	2.705	https://commons.apache.org/

**Table 2:** *Q* values of the subject software systems.

Systems	|N|	|E|	*Q*
jmeter-3.0	3339	2342	0.4292
jfreechart-1.0.19	11,946	9967	0.2429
ant-1.6.1	11,858	14,437	0.3589
struts-2.5.2	5089	3301	0.5099
freemind-1.0.1	6620	8742	0.4062
bcel-6.0	3984	3667	0.3113
mybatis-3	3193	3131	0.3131
colt-1.2.0	4735	6497	0.4650
jbullet-2.72.2.4	3288	4065	0.3603
junit4-r4.12	1576	1406	0.3277
jxtaim-0.1i	1531	1429	0.3039
commons-email-1.4	375	256	0.3422

**Table 3:** Descriptions of the subject software systems and their *Q* values.

Design Pattern	Version	LOC	|N|	|E|	*Q*
Builder	before	130	11	8	0.2656
	after	161	29	19	0.2041
Composite	before	59	10	9	−0.0787
	after	60	11	8	−0.1194
Decorator	before	34	13	6	−0.1667
	after	39	15	5	−0.1800
Iterator	before	112	4	1	0
	after	174	12	7	−0.0078
State	before	61	5	4	0.1550
	after	83	11	5	0.1200

**Table 4:** CPU time required for each step.

**Step**	**jmeter**	**jfreechart**	**ant**	**struts**	**freemind**	**bcel**	**mybatis**
i	6 s	35 s	30 s	8 s	15 s	11 s	5 s
ii	0 s	0 s	0 s	0 s	0 s	0 s	0 s
iii	0 s	0 s	0 s	0 s	0 s	0 s	0 s
**Step**	**colt**	**jbullet**	**junit4**	**jxtaim**	**commons-email**		
i	13 s	7 s	2 s	2 s	0 s		
ii	0 s	0 s	0 s	0 s	0 s		
iii	0 s	0 s	0 s	0 s	0 s		

## Supplementary Materials

The whole data sets generated and/or analyzed during the current study are available from the corresponding author on reasonable request. The sample data and our own developed software are available online at https://www.icloud.com/iclouddrive/0zRViWdWuiQkYTueyNbCIB49A#2018modularity.
