# Correction: Xiang, Y.M.; Pan, W.F.; Jiang, H.B.; Zhu, Y.F.; Li, H. Measuring Software Modularity Based on Software Networks. *Entropy* 2019, *21*, 344

**DOI:** 10.3390/e22080878

**Published:** 2020-08-10

**Authors:** Yiming Xiang, Weifeng Pan, Haibo Jiang, Yunfang Zhu, Hao Li

**Affiliations:** 1 School of Management and E-Business, Zhejiang Gongshang University, Hangzhou 310018, China; futuretech@zjgsu.edu.cn; 2 School of Computer Science and Information Engineering, Zhejiang Gongshang University, Hangzhou 310018, China; hbjiang88@163.com (H.J.); yunfangzj@163.com (Y.Z.); 3 Department of Computer Science, Western Michigan University, Kalamazoo, MI 49008, USA; hao.81.li@wmich.edu

The authors wish to make the following corrections to the paper [1].

On page 6 of [1], the last code line but one in Algorithm 1 (i.e., “18: *Q* = *sum_2m*/*sum_sigma*;”) should be updated to “18: *Q* = *sum_sigma*/*sum_2m*;”.

On page 9 of [1], the two numbers in the first line, 2.750 and 908.065, should be replaced by 2.705 and 97.880, respectively.

On page 13 of [1], the reference [8] should be replaced by the new reference [2] we listed below.

Note that the above errors do not affect the *Q* values (see Tables 2 and 3 in [1]) that we computed for each subject software system in [1], since all the *Q* values were computed according to the correct Equation (2) in [1]. Thus, the above changes will not affect the conclusions that we obtained in paper [1]. The authors would like to apologize for any inconvenience caused by these errors.
